# Online and Offline Performance Gains Following Motor Imagery Practice: A Comprehensive Review of Behavioral and Neuroimaging Studies

**DOI:** 10.3389/fnhum.2016.00315

**Published:** 2016-06-28

**Authors:** Franck Di Rienzo, Ursula Debarnot, Sébastien Daligault, Elodie Saruco, Claude Delpuech, Julien Doyon, Christian Collet, Aymeric Guillot

**Affiliations:** ^1^Laboratoire Interuniversitaire de Biologie de la Motricité, Université de Lyon, Université Claude Bernard Lyon 1Villeurbanne, France; ^2^Laboratoire de Neurologie et d’Imagerie Cognitive, Université de GenèveGeneva, Switzerland; ^3^INSERM U821, Département MEG, CERMEP Imagerie Du VivantBron, France; ^4^Unité de Neuroimagerie Fonctionnelle, Département de Psychologie, Institut Universitaire de Gériatrie de Montréal, Université de MontréalMontréal, QC, Canada; ^5^Institut Universitaire de FranceParis, France

**Keywords:** movement imagery, dynamic imagery, motor consolidation, cerebral plasticity, mental processes, sleep, motor learning

## Abstract

There is now compelling evidence that motor imagery (MI) promotes motor learning. While MI has been shown to influence the early stages of the learning process, recent data revealed that sleep also contributes to the consolidation of the memory trace. How such “*online*” and “*offline*” processes take place and how they interact to impact the neural underpinnings of movements has received little attention. The aim of the present review is twofold: (i) providing an overview of recent applied and fundamental studies investigating the effects of MI practice (MIP) on motor learning; and (ii) detangling applied and fundamental findings in support of a sleep contribution to motor consolidation after MIP. We conclude with an integrative approach of online and offline learning resulting from intense MIP in healthy participants, and underline research avenues in the motor learning/clinical domains.

## Introduction

Motor imagery (MI) is the mental representation of an action without engaging its actual execution. MI practice (MIP) refers to the repetitive use of MI to improve performance (Jackson et al., 2001). MIP research usually combines psychological and neurophysiological approaches, and represents a relevant research topic for integrative neuroscience. There is now compelling evidence that MIP positively affects motor learning, with pioneering reports dating from the first half of the 20th century (e.g., Sackett, 1934, 1935). MIP has now multiple applications in both sport sciences and rehabilitation (for an overview, see Guillot and Collet, 2008). Here, we will focus on the effects of MIP on performance in healthy individuals. Scanning the MEDLINE^®^/Pubmed^®^ database (until June 2015) through the systematic crossover of the following terms: [“*Motor imagery*”/“*Movement imagery*”/“*Mental rehearsal*”/“*Mental imagery*”/“*Mental practice*”] by [“*Performance*”/“*Learning*”/“*Sport*”] yielded 188 studies (including 30, i.e., 16% of review articles). This was thought to provide a reliable corpus to convey both the development and current trends in the field. Only interventions targeting the acquisition/improvement of motor skills were included in the pool of “motor learning” MIP articles. A related—yet distinct—research topic, since the pioneering contribution by Cornwall et al. (1991), is whether MIP can yield to force gains. Such studies primarily focused on isometric contractions, and therefore did not directly aim at improving movement kinematics. Additionally, results regarding the benefits of MIP on force remain contradictory (Guillot et al., 2010; Manochio et al., 2015).

**Figure 1:**
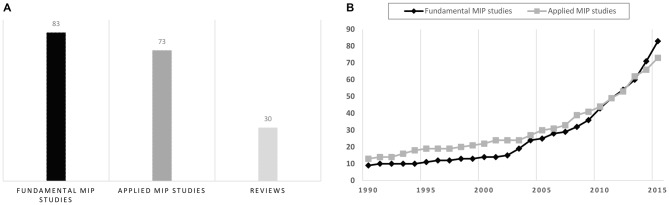
**Overview of the motor imagery practice (MIP) literature (1990–2015) based on the Pubmed/Medline^®^ database. (A)** Number of fundamental/applied MIP studies and reviews since 1990. **(B)** Cumulated number of fundamental and applied MIP studies from January 1990 to June 2015. The increase in number of functional brain imaging investigations paradigms carried out since 2000, which was due to the emergence of fundamental research topics addressing the neurophysiological underpinnings of MIP effects on motor performance, explains why fundamental studies progressively outnumbered applied MIP studies.

Overall, MIP articles included both applied and fundamental motor learning studies (Figure 1). Applied MIP studies followed a pragmatic approach, and primary aimed at assessing MIP efficacy at the behavioral level. Interventions were delivered in the actual context of a specific sport/professional discipline (e.g., music, sports, surgery, etc.). Fundamental MIP studies additionally addressed research issues related to the psychophysiological underpinnings of the hypothesized effects on learning. Further, these studies frequently considered simple movements (typically single-joint actions) performed in standardized laboratory contexts. MIP studies published before the 1990s almost exclusively belong to the field of sport psychology. These have been elegantly summarized in seminal review articles and meta-analyses (Feltz and Landers, 1983; Driskell et al., 1994). MIP studies since 2000 include a larger proportion of fundamental studies, with an increase in functional brain imaging investigations intended to delineate the psychophysiological processes underlying MIP efficacy. Fundamental studies thus progressively outnumbered applied MIP studies (Figure 1). Fundamental findings on the psychophysiological underpinnings of MIP should ideally guide applied research (e.g., new domains of applications, optimal conditions of practice, etc.,). Yet, the field in fact progressively evolved from applied to more fundamental research. To convey how the field developed during the last decades, we chose to first discuss applied, and then fundamental findings, in the forthcoming sections.

Motor learning is classically defined as a change in motor behavior resulting from practice. Accordingly, motor learning is quantified in terms of performance improvements before and after a practice intervention in longitudinal research designs. When the practice intervention involves multiple sessions within a span of several days/weeks, the cumulated effects on performance are evaluated to attest motor learning. These can be summarized as *online* learning processes, since they occur as a *direct* consequence of practice. Several authors underlined in conceptual frameworks that motor learning cannot be considered a linear process of performance improvement (e.g., Yelle, 1979; Mayer-Kress et al., 2009). For instance, Doyon and Benali (2005) highlighted the involvement of functional interactions between cortico-striatal and cortico-cerebellar brain systems during the early stages of motor learning, i.e., corresponding to the rapid performance improvements consecutive to a single/a series of practice session(s). The automatization stage of motor learning, corresponding to slower performance improvements yielding to increased motor efficiency, involved to a greater extent the cortico-striatal system (Doyon and Ungerleider, 2002). While learning stages differ in terms of behavioral/neurophysiological correlates, they commonly result from online learning processes. Doyon and Benali (2005) also acknowledged the consolidation stage, characterized by delayed performance gains occurring after a latent period of approximately 6 h, in the absence of additional practice. These can be summarized as* offline* learning processes, since they *indirectly* result from practice. Performance improvements consecutive to a night of sleep is a well-established correlate of offline learning (Brashers-Krug et al., 1996; Karni et al., 1998). Delayed/spontaneous performance improvements are also sensitive to motor interferences (e.g., Korman et al., 2007). Practically, delayed performance gains and robustness to interference are two important behavioral correlates of offline learning processes (for a review see Krakauer and Shadmehr, 2006), and should thus be considered concurrently when investigating whether a period of sleep contributes to enhance motor performance. Former review articles considered performance improvements immediately resulting from MIP interventions (i.e., MIP effects on *online* learning processes). Surprisingly, they did not consider the potential *delayed* performance gains consecutive to MIP, in other words the MIP effects on offline learning. The present review was therefore designed to provide a comprehensive overview of motor learning after MIP in healthy participants in relation to both online and offline processes.

## Online Learning Processes

### Applied Studies

#### Effect on Quantitative and Qualitative Indexes of Performance

**Figure 2:**
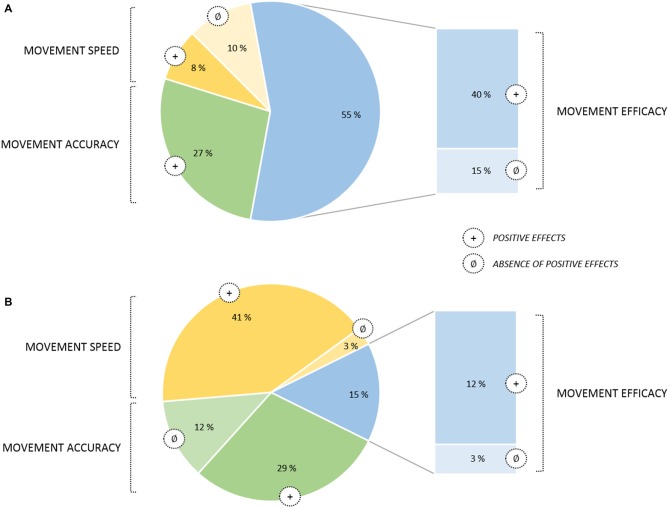
**Pie chart of movement parameters affected by online learning through MIP (based on a sample of 122 studies published from January 2000 to June 2015). (A)** Graph for applied MIP studies (*n* = 52). **(B)** Graph for fundamental MIP studies (*n* = 70). Improvements quantified in terms of movement efficacy are displayed in a separate sector of the chart since this category involved a broader set of motor performance indexes. Movement efficacy encompassed both objective (e.g., movement coordination, success rate) and subjective (e.g., scale ratings on the technical execution) criterion. Noteworthy, no deleterious effects of MIP were found. Also, while for applied studies MIP efficacy on movement speed yielded contradictory results, with positive effects were almost systematically reported in fundamental studies. A reversed pattern of results emerged for movement accuracy, with positive effects being systematically reported in applied MIP studies but less consistently in fundamental studies.

From a conceptual viewpoint, there has been a great deal of research on imagery processes for well over a century (Kosslyn et al., 2006), and there is now ample evidence that MIP can substantially contribute to promote motor learning. In the sport domain, MI is very popular among athletes and coaches, and has been described as a “*Centre pillar of applied sport psychology*” (Morris et al., 2005; Cumming and Williams, 2013). As mentioned previously, there has been an important number of relevant reviews and meta-analyses focusing on the benefits of MIP (Feltz and Landers, 1983; Driskell et al., 1994; Holmes and Collins, 2001; Guillot and Collet, 2008; Murphy et al., 2008; Weinberg, 2008; Schuster et al., 2011; Cumming and Williams, 2013; Rao et al., 2015). All focused on MIP findings attesting positive effects on online learning processes. This yielded multiple practical applications and theoretical models. Among them, Guillot and Collet (2008) distinguished four main imagery outcomes in their model (Motor Imagery Integrative Model in Sport), covering the main practical applications of MIP: *(i)* Motor learning and Performance; *(ii)* Motivation, Self-confidence and Anxiety; *(iii)* Strategies and Problem-solving; and *(iv)* Injury Rehabilitation. Overall, particular attention has been paid to the effect of cost-effective MIP interventions in enhancing online learning, MIP improving both quantitative and qualitative aspects of the motor performance (Figure 2).

MIP was first shown to enhance movement accuracy. For instance, Guillot et al. (2015) showed that embedded MIP blunted the decrease of subsequent tennis shot accuracy usually observed during high intensity interval training sessions, hence preserving the level of performance during intense practice. Afrouzeh et al. (2015) also reported greater pass accuracy in volley-ball players after MIP. A second set of applied studies provided strong evidence that MIP is likely to impact movement speed. Boschker et al. (2000) first reported that increasing or decreasing MI speed of a motor sequence might elicit comparable changes in actual movement speed. They investigated the effect of mentally or physically performing a sequence of 12 rhythmic basic steps at a slow/fast pace, and provided evidence that changing MI speed resulted in similar modifications of the actual speed during a subsequent retention test. Louis et al. (2008) confirmed that MI might affect the execution time of subsequent motor tasks, even in highly automated sport actions. Using sequential finger movement sequences, Debarnot et al. (2010) and Avanzino et al. (2009) reported that MIP, either performed in real time or at a faster pace, was likely to increase movement velocity, particularly for the most complex sequences (i.e., bimanual). Although such effects of MI on actual movement speed are not systematic (O and Munroe-Chandler, 2008), and even though decreasing MI speed to correct and adjust fine visual-motor tasks might be beneficial during the early stages of learning (O and Hall, 2009), this may be frequently detrimental to achieve expert performance—where accurate timing is seminal. Surprisingly, there is yet no experimental data examining the effect of MIP on actual movement speed which controlled, concomitantly, the possible alterations of the technical execution. Concluding about the effects of changing MI speed might thus be premature before ensuring that movement efficacy is not altered.

Finally, MIP was found to improve movement efficacy. This is reflected through both objective and subjective evaluations which addressed qualitative/quantitative aspects of the motor performance (e.g., scoring performance in a given discipline, technical realization). Overall, there is accumulated evidence that MIP contributes to achieve a greater level of sporting performance (Schuster et al., 2011; Guillot et al., 2013a; Williams et al., 2013), or through a subjective/qualitative appreciation of movement efficacy (Arora et al., 2011; Guillot et al., 2013b). Furthermore, MIP was shown not only to improve the overall performance, but also to impact specific movement kinematics. For instance, Battaglia et al. (2014) reported that both the flight time and the ground-contact time were significantly improved during performance of the Hopping and Drop Jump tests, after a mental training program in national rhythmic gymnasts. Likewise, Giron et al. (2012) provided evidence that MIP contributed to enhance pelvis and hip kinematics during dance movements, with visual and kinesthetic imagery leading to distinct peak external hip rotations. Olsson et al. (2008b) further reported that MIP might specifically improve some technical components of complex motor tasks (i.e., high-jump). The authors investigated the efficacy of an internal imagery intervention in active high jumpers by measuring four appropriate outcome measures of performance: jumping height, number of false jumps, take-off angle, and bar clearance (i.e., the virtual line-distance from the foot to the shoulder when the athlete is over the bar). Data revealed a significant improvement on bar clearance only, which is the most complex technical component of the motor sequence. Such findings confirm that researchers should not only pay attention to the final performance, but also consider technical outcome measures. This conceptual approach of performance analysis is of importance, as improving bar clearance might result in higher jumping height over time, even in the absence of immediate positive effects.

#### Practical Implications

Both the theoretical accounts of MI use and the experimental data designed to determine the best way to perform MI adequately cover the main key-components that need to be carefully controlled to ensure the effectiveness of MI to achieve greater motor performance. Several theoretical models and MI frameworks have been designed to support efficient MI interventions (e.g., Holmes and Collins, 2001; Guillot and Collet, 2008), enabling researchers to infer optimal MIP guidelines across several disciplines requiring motor expertise (for a systematic review see Schuster et al., 2011). This approach, which is nicely and extensively illustrated in the imagery literature, will not be developed in the present review. Interestingly, there is a substantial overlap of active brain regions during MIP and physical practice of the corresponding movement (for exhaustive reviews see Munzert et al., 2009; Guillot et al., 2012a; Hétu et al., 2013). Efficient forms of MIP may strongly engage the motor systems to increase the connectivity between motor system regions. MIP should thus be more efficient if it involves the same processes than those engaged while preparing, programming and controlling actual movements (see “Theoretical Implications” section, for further development). While common brain networks are activated during both physical practice and MI of the same task, and as there is no actual feedback during MI, an important question remained to determine how adequately combining these two forms of practice and the optimal ratio of physical vs. MIP.

Courtine et al. (2004) demonstrated the superiority of alternating MI and physical practice compared to performing a single block of MI trials, as shown by a significant decrease in timing variability. A recent study by Rozand et al. (2015) further showed that performing a prolonged session of MI without any sensory feedback might be harmful, but including regular physical execution trials contributed to reduce the sensation of mental fatigue and prevented from the alteration of actual and imagined movement durations. Interestingly, Allami et al. (2008) examined the selective efficacy of different ratios of physical to MIP. Overall, data revealed that performing MI at high rates (e.g., 50–75%) along with physical practice might result (at least) in comparable levels of performance compared to physical practice alone. A similar finding was reported by Sanders et al. (2004), who investigated the benefits of MIP in medical students learning basic surgical procedures. They concluded that MI might be as effective as PP once students have received adequate instructions and followed a monitored physical practice session beforehand.

When considering the place of MIP in mental training programs, another promising avenue is its combination with action observation (for an extensive review see Vogt et al., 2013). While the effects of action observation and MI have been extensively studied and documented in isolation from each other, Vogt et al. (2013) recently proposed an interesting spectrum ranging from congruent to conflicting action observation and MI coupling, in order to probe the two component processes. Results from recent neuroimaging and electrophysiological studies have confirmed that combining MI and action observation might result in enhanced cortical and subcortical activations relative to each form of practice alone, in regions of interest including the motor systems and the parietal areas (Macuga and Frey, 2012; Nedelko et al., 2012; Berends et al., 2013; Villiger et al., 2013; Taube et al., 2015). A substantial overlap is also observed when comparing combined action observation/MI with action execution, hence supporting the degree of functional equivalence and both the immediate facilitative and longer-term positive effects of coupling these techniques (Taube et al., 2014, 2015). Therefore, instead of contrasting the respective benefits of action observation and MIP on motor (re)learning, the best training effects might be expected by combined MI/action observation practice. Such mental training procedures might yield to a higher level of functional equivalence and potentiate the stimulation.

A debated point of consideration is the intrinsic nature of the MI work, and how it relates to physical practice. The static/dynamic distinction of imagery processes has been early considered by researchers. Paivio and Clark (1991) provided a comprehensive review of how one can imagine stationary objects, but also objects in motion or being rotated and transformed. This conceptualization refers to the perception of movement during MI of objects with a dynamic quality, or images of objects being transformed and manipulated. Since these studies, however, the dynamic properties of MI no longer characterize the symbolic representation of movements and transformations. A second and more practical consideration of static/dynamic imagery considered whether participants were moving or remained motionless during MI. According to Gould and Damarjian (1996), however, replicating the actual movements during MI, while holding a piece of equipment relevant to the sport/situation, might contribute to facilitate and increase the efficacy of MIP. We all have in mind pictures of athletes moving while imagining their subsequent performance during pre-performance routines, which challenges the traditional assumption that MI requires the athlete remaining motionless. The fact that athletes often move slightly while engaged in MI has therefore spawned interest in MI research. Experimental studies showed that such dynamic imagery might contribute to increase the vividness and temporal accuracy of MI (Callow et al., 2006; Guillot et al., 2013b; Fusco et al., 2014). As initially suggested by Gould and Damarjian (1996), who proposed that dynamic imagery promotes the recall of the sensations associated with the actual performance, we state that moving while imagining may prime and facilitate the MI experience based on the actual feedback, and therefore contribute to improve subsequent motor performance. This might also improve temporal congruence by emphasizing the degree of behavioral matching, and possibly enhance the functional equivalence between MI and motor performance (van der Meulen et al., 2014). Interestingly, Ferreira Dias Kanthack et al. (2016) investigated whether the benefits of dynamic over static MI remained effective under physical fatigue. They showed that the optimal use of static and dynamic MI may be linked to exhaustion/energy expenditure, as dynamic MI was superior to static MI to improve movement accuracy when athletes were not fatigued. In contrast, static MI remained more efficient to enhance performance under physical fatigue. They argued that the current physical state might affect the body representation, so that performing dynamic MI under fatigue may create interferences between actual and predicted body states (Demougeot and Papaxanthis, 2011). Dynamic MI might therefore be prioritized in the absence of fatigue, while static MI should be preferred under fatigue state. Based on these data, we state that dynamic imagery should incorporate slight congruent movements to enhance the process, but the amplitude of these movements should be carefully defined to avoid a misunderstanding between MI and motor performance. We therefore propose to define dynamic MI as:

*“A type of MI where athletes adopt a congruent body position and embody spatial and/or temporal invariants of the movement without entirely performing it”*.

Conceptually, performing dynamic imagery is different from imagining while moving by engaging the full body in the action. The latter form has received less attention and is not common, even though athletes can punctually form mental representations during physical practice (Van Gyn et al., 1990; Hanrahan, 1995; Nordin and Cumming, 2007). For instance, Vergeer and Roberts (2006) investigated the efficacy of MIP during stretching on flexibility gains, imagery vividness, and perceived comfort. While there was no significant effect on performance, they reported a positive effect on the perceived comfort. More recently, Kanthack et al. (2016) examined the short-term effects of MIP during a stretching exercise, with a specific focus on its effects on muscle and autonomic nervous system responses. They reported reduced muscle activation allowing a more effective stretch of the connective tissues, hence eliciting significant stretching performance gains. Taken together, these data provide evidence of the benefits of using MI during movements, even though it challenges the common belief that MI occurs in the absence of sensory input. As outlined by MacIntyre and Moran (2010), performing dynamic imagery and/or using MI during actual practice requires reconsidering our theoretical conceptual definitions of MI.

### Fundamental Studies

#### Effects on Neural Plasticity

There is a general consensus that experience-dependent changes in motor behavior originate from structural and/or functional reorganizations in the connectivity of neurons, i.e., activity-dependent neuroplasticity (for reviews see Salmon and Butters, 1995; Sanes, 2003; Ioffe, 2004; May, 2011). Empirically, the assumption that MIP could induce activity-dependent neuroplasticity has been early considered (e.g., Warner and McNeill, 1988). This postulate was driven by: *(i)* motor learning experiments attesting MIP efficacy (behavioral changes being hypothetically grounded in parallel neurophysiological adaptations to those underlying the effects of physical training); and *(ii)* functional brain imaging findings supporting the functional equivalence principle. Accordingly, MI and physical practice of the corresponding action engage both overlapping neural networks and comparable patterns of connectivity between brain motor system regions (e.g., Lotze and Halsband, 2006; Munzert et al., 2009; Gao et al., 2011). Peripheral neurophysiological recordings of somatic and autonomic activities have further established a solid scientific background supporting that physical practice and MI belong to the same action-state continuum (for reviews see Stinear, 2010; Guillot et al., 2012a; Collet et al., 2013). This is in keeping with the early postulate by Stephan and Frackowiak (1996), who considered MI as an intermediate motor behavior between the cognitive motor processes and the physical performance of an action. MI would thus represent an efficient method to stimulate brain motor networks mediating skill acquisition (for recent insights, see Kraeutner et al., 2014).

**Figure 3:**
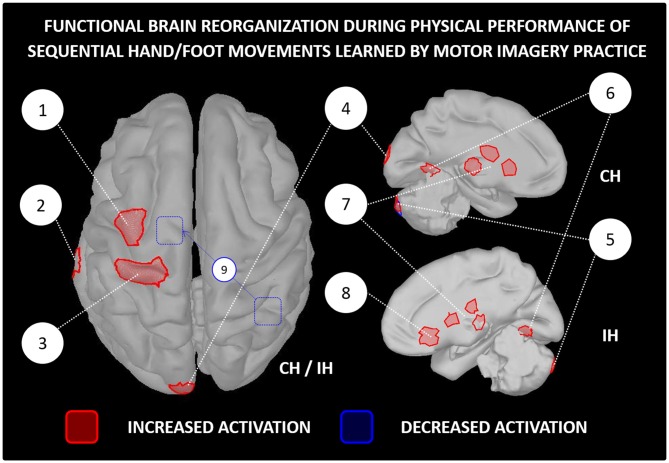
**Functional reorganization of the brain networks controlling the physical performance of a motor task learnt by MIP *only*.** The figure is based on functional brain imaging experiments which performed source reconstruction analyses. Only paradigms involving sequential hand/foot movements met such inclusion criteria (e.g., Jackson et al., 2003; Lacourse et al., 2004; Nyberg et al., 2006; Zhang et al., 2011, 2012). Functional brain imaging experiments assessing neuroplasticity following MIP by examining brain activations *during MI* were not included (e.g., Sauvage et al., 2015). 1-Premotor cortex, 2-Middle temporal gyrus, 3-Primary motor cortex, 4-Occipital cortex, 5-Cerebellum, 6-Fusiform gyrus, 7-Thalamus and basal ganglia (caudate nucleus and putamen), 8-Orbitofrontal cortex, 9-Decreased functional connectivity between the right inferior parietal lobe and the supplementary motor area after MIP. MIP, Motor imagery practice; CH, Contralateral hemisphere; IH, Ipsilateral hemisphere.

While scientific evidence of activity-dependent neuroplasticity is accumulating in the field of brain computer interfaces and neurologic rehabilitation (Mokienko et al., 2013; Di Rienzo et al., 2014; Ahn and Jun, 2015), scientific reports of learning-dependent brain changes after MIP in healthy participants remain somehow limited. Pascual-Leone et al. (1995) provided a pioneering straightforward evidence of activity-dependent neuroplasticity consecutive to MIP. Using transcranial magnetic simulation, the authors observed an enlargement of the cortical representation of hand muscles controlling a piano sequence learned by MI (2 h of practice per day during 5 days). The cortical changes were identical in the MIP and physical training groups, although physical training outperformed MIP in terms of performance improvements. Interestingly, the adjunction of a single physical practice session in the MIP group enabled to reach a similar level of performance. The authors suggested that while MIP prompts activity-dependent neuroplasticity at the brain level, physical practice facilitates the actualization of the central changes at the behavioral level (stabilization of labile reorganizations). Accordingly, for simple motor tasks, MIP may replace up to 75% the physical training if a minimal ratio of physical practice is delivered to compensate the deficits in performance improvements (Allami et al., 2014). In reference to the principle of functional equivalence, and in the same vein of Pascual-Leone et al. (1995), Jackson et al. (2003) hypothesized that MIP would induce learning-dependent brain changes comparable to those observed after physical practice, and that such changes would be measured during both physical and mental performance. Based on a sequence of foot movements learnt over the course of 1 week (5 MIP sessions), functional brain imaging data with positron emission tomography confirmed the main hypotheses. Increased contralateral orbitofrontal cortex and reduced ipsilateral cerebellum activations were recorded in the MIP group, but not in the control group. These brain changes corresponded to those elicited after physical practice of the same task, as reported in an earlier study (Lafleur et al., 2002). Findings of: *(i)* reinforcement of brain activity within motor system regions (i.e., more intense and focused activations, sometimes with reduced recruitment of associative regions, Figure 3); and *(ii)* preservation of functional equivalence between MI and physical practice after motor learning (i.e., learning-dependent changes being reflected in brain activations during both physical and MI) were later replicated in several experiments (e.g., Lacourse et al., 2004; Nyberg et al., 2006; Olsson et al., 2008c; Zhang et al., 2011), in spite of the different nature of the motor tasks across protocols (e.g., sequential hand/foot movements, locomotor abilities).

The effects of MIP on activity-dependent neuroplasticity in longitudinal designs have not only been observed as participants physically performed the task learnt, but also as they imagined it before and after a MIP program. For instance, Sacco et al. (2006) administered a 5-day MIP intervention embedded within classical tango dance lessons, to emphasize the attentional control of locomotion in participants without any prior dance experience. During the post-test, the authors observed increased activation of the bilateral primary sensorimotor and left parietal cortices, with concomitant decrease of cerebellar activations during MI of walking. In a more fundamental approach, Sauvage et al. (2015) observed reduced fronto-parietal activations and increased cingulate/basal ganglia recruitment during MI of a sequence of foot movements learnt by MIP over a 1 week period (five sessions of 100 MI trials). Notably, transversal studies examining the neural networks controlling MI in novices and expert athletes/professionals emphasized long-term brain reorganizations mediating expertise. The most recent experiments reported differences in the resting state brain networks after MIP intervention. Particularly, these experiments emphasized increased connectivity between regions of the brain motor system, rather than differences in resting state levels of activation (Zhang et al., 2014; Ge et al., 2015). These data therefore suggest that MIP leads to large-scale functional reorganizations of the motor networks, which can be assessed from various brain states.

Recent findings keep extending the knowledge regarding the effects of MIP on online learning processes. For instance, in addition to classical brain activation contrasts, functional connectivity measures brought further knowledge regarding how MIP affect the functional interplay between brain motor regions. Using graph theory analyses, Zhang et al. (2012) observed that learning effects during a finger tapping sequence in the MIP group (2 weeks of practice, 30 min of practice per day) reduced the connectivity of the ipsilateral posterior parietal cortex with cortical/subcortical regions of the motor network, notably the SMA, during both actual and imagined performance. Such changes were absent in the no-learning control group, and thus potentially reflected a more efficient allocation of mental resources to complete the task after MIP. Additionally, brain stimulation paradigms demonstrated their efficacy to facilitate or interfere with the effects of MIP on motor learning. For instance, Transcranial Magnetic Stimulation (TMS) applied over the inferior parietal lobe interfered with implicit learning of a sequential button-press task (Kraeutner et al., 2015). Conversely, applying transcranial direct current stimulation to the primary motor cortex during MI increased its beneficial effects on the online learning of a finger tapping sequence (Saimpont et al., 2015). Previously, Foerster et al. (2013) reported similar findings on writing skills using transcranial direct current stimulation of the primary motor cortex and dorsolateral prefrontal cortex. Yet, these paradigms did not include a physical training condition (with or without brain stimulation). Nonetheless, they adopted a radically different use of electromagnetic brain stimulations compared to the early neurophysiological MIP studies. Brain stimulation techniques were primarily used to assess brain changes after MIP (e.g., Pascual-Leone et al., 1995; see also Avanzino et al., 2015 for a recent TMS investigation of primary motor cortex neuroplasticity). Fundamental MIP experiments on healthy participants frequently put their findings in the perspective of clinical applications, albeit the guidelines for efficient MIP with clinical populations may vary to a great extent compared to those in healthy participants (Di Rienzo et al., 2014). In this vein, recent approaches attempted to evaluate *a priori* the clinical efficacy of MIP (and their neurophysiological basis) from data measured in healthy participants. Particularly, Volz et al. (2015) studied whether a single session of MIP (20 min of finger-to-thumb oppositions) decreased the pain threshold evoked by thenar pressure (see Einsiedel et al., 2011; Frenkel et al., 2014 for a similar approach in a clinical model of joint immobilization). The authors measured reduced pain threshold in the MIP group, but not in control subjects. The changes in pain perception were correlated to decreased corticospinal excitability in the efferent pathways targeting the thenar during voluntary contractions, which may have implications for patients suffering from chronic pain.

The positive effects of MIP on neuroplasticity in motor performance paradigms may not be systematic. For instance, Bassolino et al. (2014) observed that, contrary to action observation, MIP of grasping exercises failed to prevent the corticomotor depression caused by 10 h of arm immobilization in healthy subjects (i.e., reduction of the corticomotor map of the first dorsal interosseus evoked by TMS). Unfortunately, the experimental paradigm did not involve any behavioral measures. While the authors concluded that MIP was inefficient to prevent corticospinal depression after immobilization (for an opposite pattern of results of MIP and action observation on corticospinal excitability, see Bianco et al., 2012), this lack of behavioral control is somehow problematic as the results contradict several experiments attesting at a behavioral and/or neurophysiological level the efficacy of MIP to limit the deleterious effects of immobilization on joint range of motion (Einsiedel et al., 2011; Frenkel et al., 2014). The number of experiments investigating activity-dependent brain changes in healthy participants after MIP increases on a regular basis since 2000, hence reflecting the consideration of neuroscientists for the method. Future research should highlight new factors which may influence the outcome of MIP interventions, thereby explaining divergent results. A recent work by Herholz et al. (2015) underlined the issue of individual profiles of responsiveness to MIP. In a piano-sequence learning paradigm, the authors detangled the neurophysiological correlates of the inter-individual predispositions to benefit from MIP. Before the intervention, participants who exhibited the highest activation intensities in the primary auditory cortex and hippocampus (while listening to the piano sequence), and in the premotor cortex and thalamic regions (while imagining the piano sequence), achieved the highest learning rates. Notably, reduced activations in frontal and occipital cortices (as well as in the precuneus) were also significant predictors of the learning rate. Future research on the neurophysiological correlates of individual predispositions towards MIP effects on activity-dependent neuroplasticity may enable to adjust MIP intervention frameworks to optimize their efficacy and potentially account for contradictory results related to the efficacy of some interventions.

#### Theoretical Implications

Until the end of the 20th century, the effects of MIP on online learning processes were attributed to psychological and/or cognitive factors (Kohl and Roenker, 1983). For instance, the “*Symbolic learning*” theory by Sackett (1934) proposed that mental rehearsal involved a specific focus on symbolic components such as the spatial and/or temporal invariants of the movement (due to the absence of actual motor output). This was assumed to facilitate cognitive processing during the forthcoming task performance. These theories of MIP were emphasized in early reviews that focused on MIP and online learning (Feltz and Landers, 1983) as an account of higher benefits of MIP on online learning of skills requiring a high cognitive demand (Driskell et al., 1994). Another classification of MIP use was based on the 2 × 2 conceptual framework by Paivio (1985). MI was assumed to impact both cognitive and motivational functions and to operate on general and specific levels. This resulted in four functions of MIP. Hall et al. (1998) extended this model by subdividing the motivational-general function into motivational general-arousal and motivational general-mastery sub-modalities. Overall, such classifications support a contribution of MIP to improve motor performance by driving focus on psychological factors such as strategies and routine, self-achievement, arousal/affect, self-confidence and mental toughness (for an extensive review see Cumming and Williams, 2013).

The seminal contribution of M. Jeannerod, referred to as the “*simulation theory*” (e.g., Jeannerod, 2001) conceptualized MI as an inhibited form of voluntary motor behavior (see also Jeannerod and Decety, 1995; Jeannerod, 1995). According to this framework, MI is a conscious access to the content of the motor preparation. The motor preparation would be emulated into a sensory experience due to its active inhibition during mental rehearsal: “*If motor preparation (…) could be prolonged, the intention to act would become progressively a MI of the same action (…). Actions which fail or which are cancelled at the last moment may be situations where a non-conscious program is transformed into a conscious image*” (Jeannerod, 1994, p. 7–8). Gandevia et al. (1997) argued, in the same vein, that MIP facilitates neural processing within the neural circuits controlling the action, due to subliminal activation of the somatic pathways. These theories of MIP share the postulate that MIP improves performance through the preliminary rehearsal of psychological/cognitive/neurophysiological components, which exerts a preparatory effect on the actual performance. MIP effects on performance would thus reflect “*priming effects*”, namely: *“(…) A type of implicit learning wherein a stimulus prompts a change in behavior”* (Stoykov and Madhavan, 2015, p. 1).

These approaches are obviously sound and scientifically grounded. They may be extended at the scope of recent evidence that MI not only engages the psychophysiological processes involved during motor preparation but also those mediating the actual execution. Functional brain imaging demonstrated that MIP stimulates both premotor and primary sensorimotor brain structures (for recent insights, see Gemignani et al., 2004; Burianová et al., 2013; Kraeutner et al., 2014)^1^. Functional brain imaging evidence of activity-dependent brain reorganizations consecutive to MIP is accumulating (Figure 3). Assuming that both short- and long-term effects of MIP on motor performance are mediated by activity-dependent neural reorganizations (e.g., short-term changes in synaptic gain and/or long-term scaling of labile networks through stabilization of latent synapses), a neural plasticity approach of MIP effects would represent a unified framework to explain/interpret the positive results of MIP on online learning processes (for pioneering insights, see Decety and Ingvar, 1990). It is worth mentioning that this postulate derives from findings yielded by explicit online learning paradigms, where participants focused on a specific movement during MIP. Original findings by Kraeutner et al. (2016) revealed that MIP could also promote implicit learning of sequential movements (see Ingram et al., 2016 for recent insights regarding the nature of implicit learning through MIP, as revealed by transfer/interference conditions). TMS data further revealed that inhibiting parietal structures prevented implicit learning (Kraeutner et al., 2015). Detangling the neurophysiological correlates mediating implicit vs. explicit online learning through MIP thus represents a novel and exciting research issue. Finally, the postulate that MIP efficacy is grounded in activity-dependent neural reorganizations provides a neurophysiological rationale to the practical guidelines supporting efficient MIP. For instance, practicing MI in an environmental context and according to sensory modalities matching those encountered during physical practice contributes to reduce the “*subjective distance*” (Jeannerod, 1995) between overt and covert motor performance, which in turn enhances recruitment of brain motor areas (e.g., Fourkas et al., 2008; Lorey et al., 2009; Mizuguchi et al., 2013; Bisio et al., 2014; Wang et al., 2014).

## Offline Learning Processes

### Applied Studies

Despite some challenging results (Rickard et al., 2008; Nettersheim et al., 2015), sleep has been shown to play a critical role in the consolidation of motor performance after physical practice (Stickgold and Walker, 2005; Doyon et al., 2009; Albouy et al., 2013b), as well as action observation (Van Der Werf et al., 2009). Yet, looking for similar effects following MIP has received little attention but showed promising results. However, experimental studies looking at this issue only investigated whether a period of sleep contributed to delayed performance gains for simple movements performed in a standardized laboratory context. There is therefore no real applied studies exploring offline learning processes according to the theoretical definition of applied vs. fundamental studies adopted for the present review. Such line of research is of practical interest in the motor learning and clinical domains, but preliminarily requires fundamental studies providing strong evidence of the benefits of sleep after MIP, and determining the neural underpinnings of such offline learning effects.

### Fundamental Studies

Based on the functional equivalence between MI and actual motor performance, offline performance gains following MIP might be expected during sleep, as it has been established for physical practice. First evidence of such effects comes from studies in which healthy participants performed either a motor adaptation task (requiring compensating the movement for environmental changes, Doyon and Benali, 2005; Hardwick et al., 2013), a motor sequential learning task, or a mental rotation task, before and after a night of sleep (Debarnot et al., 2009a,b, 2013). In all cases, data revealed the existence of substantial sleep-related gains following MIP. Interestingly, there was no correlation between the measure of underestimation of the time to imagine the motor sequence, which is likely to affect the MI quality (Louis et al., 2008; Guillot et al., 2012b), and actual speed gains after sleep. These results provided evidence that sleep contributes to motor memory consolidation after MIP, and further suggested that offline delayed gains are not related to the intrinsic characteristics (e.g., speed) of MI. As shown by Kuriyama et al. (2004) for actual practice, Debarnot et al. (2012a) later demonstrated that the most complex sequential finger movements to be imagined were the most effective in promoting sleep-related performance gains, with larger overnight improvement for movements involving bimanual coordination. These findings support that delayed performance gains for imagined movements partially depend on motor skill complexity. Analyses of the transitions between the elements of the motor task further revealed greatest speed enhancement for the most difficult transitions. In a more recent study, Debarnot et al. (2015) compared the effects of variable and constant MIP on the acquisition, consolidation, and transfer of visuomotor sequential learning. Data revealed significant delayed performance gains after variable MIP compared to both constant MI and the simple passage of daytime, hence providing new insight in the scheduling and content of MI sessions. Interestingly, not only a night of sleep, but also daytime naps were found to facilitate the motor memory consolidation of imagined movements, compared with spending a similar time interval in the awake state (Debarnot et al., 2011). Delayed performance gains were observed regardless of the nap duration, i.e., after short naps including 10 min of stage 2 sleep or long naps of 60–90 min period including slow-wave and rapid eye movement sleep. This result highlights the importance of non-rapid eye movement sleep including the stage 2 for efficient motor consolidation (Nishida and Walker, 2007; Morin et al., 2008; Albouy et al., 2013a).

Besides delayed gains in performance (Korman et al., 2007), the susceptibility to retrograde interference (disruptive effect of a later experience on the consolidation in memory of a prior training experience) should also be considered (Krakauer and Shadmehr, 2006). Yet, only Debarnot et al. (2010) examined the effect of a retroactive motor interference (administered 2 h after MIP) on motor consolidation after a night of sleep. As in Korman et al. (2007), they showed that performing a motor interference task prevented the expression of delayed gains at 24 h post-physical training, while practicing the first motor learning through MIP followed by the physical interfering task did not alter the motor consolidation process (Debarnot et al., 2010). This result highlights the relevance of a period of sleep for motor consolidation after MIP, and further supports that MIP might result in a durable and flexible representation of task requirements (Wohldmann et al., 2008). Moreover, this finding suggests that MIP may occasionally be a better alternative to consolidate motor skills than physical practice, by strengthening an abstract representation that does not involve specific effectors. Interestingly, in contrast to such procedural motor interference, Debarnot et al. (2012b) later showed that a declarative interference task might affect the offline motor consolidation following MIP. Data revealed that declarative interference (i.e., word-list task) altered overnight and daytime consolidation of MIP learning, but with delayed gains in performance still occurring after a night of sleep compared to wakefulness. In other words, sleep compensated the detrimental effect of declarative interference, unlike wakefulness. Surprisingly, a last issue that has been neglected in the current literature is the potential (lack of) retrograde interference of a secondary MI task on the motor consolidation of a first motor task also learnt through MIP. Future studies will certainly consider this retrograde influence and contribute to better understand the effects of MIP on motor consolidation.

Spurred by the data mentioned above, and albeit this line of research is quite recent, combining sleep and MIP in motor learning protocols is a promising avenue. From a more theoretical viewpoint, determining the neural processes underpinning the need for sleep to consolidate motor memories after MIP, as well as the factors susceptible to limit benefits of sleep, are questions currently under consideration. Yet, whether brain plasticity observed during MI is later reactivated during the period of sleep following MIP, as shown for physical practice (e.g., Stickgold and Walker, 2007), needs to be addressed. Likewise, future research should better determine the stages of sleep that are critical for discrete steps in motor consolidation following MI. As for motor skill consolidation, there may be more than a single phase of sleep-dependent consolidation. In particular, as sleep-spindle activity is thought to play a critical role in motor consolidation by facilitating the neuronal plasticity (Barakat et al., 2011; Albouy et al., 2013b), further investigations including recording sleep-related polysomnographic data after MIP are required.

## Conclusion

**Figure 4:**
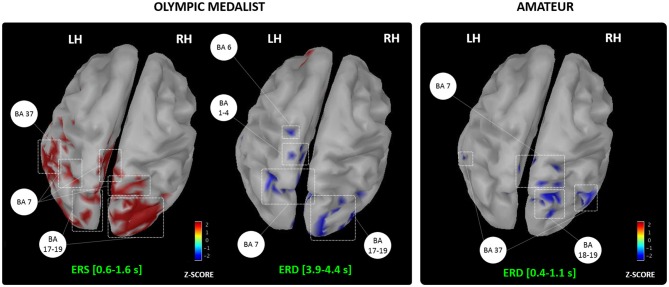
**Generators of the alpha event-related synchronization/desynchronization recorded in an Olympic and amateur athletes during MI of the snatch.** BA, Brodmann areas; LH/RH, Left/Right hemispheres; ERS, Event-related synchronization; ERD, Event-Related desynchronization. Adapted with permission from Di Rienzo et al. (2016).

We reviewed the effects of MIP on both online and offline learning processes in healthy participants. Activity-dependent neuroplasticity resulting from MIP is a plausible origin to online learning effects assessed at a behavioral level (e.g., movement accuracy, movement speed and movement efficacy, Figure 2). Yet, the neurophysiological correlates of MIP on offline learning processes remain unexplored. Overall, MIP can facilitate access to motor expertise, which can be considered the long-term result of successive online and offline learning processes. Interestingly, motor expertise, in turn, yields to activity-dependent neural reorganizations of brain networks controlling both actual and imagined performance. The imagery literature provided ample evidence of such reorganizations across various disciplines (Olsson et al., 2008a; Sacco et al., 2009; Wei and Luo, 2010; Chang et al., 2011; Baeck et al., 2012; Bezzola et al., 2012; Olshansky et al., 2015; Wolf et al., 2015), hence attesting that brain activations during MI reflected life-long brain changes resulting from successive online and offline neural reorganizations elicited by intense amounts of practice. Brain activity during MI reflects the motor automatization taking place along the course of development (Cebolla et al., 2015), but also mirrors expertise-dependent changes in the brain networks of *athletes* (for a review see Debarnot et al., 2014). Nonetheless, past studies on expertise-dependent changes of MI networks rarely compared two *extreme* levels on the expertise continuum, namely an Olympic level champion vs. a novice athlete. The study by Di Rienzo et al. (2016) may be an original and informative illustration of such contrast to punctuate this review. Using magnetoencephalography (MEG), they gained access to the generators of mu desynchronization during the representation of MI of a snatch in an Olympic weightlifting athlete and a novice participant competing at a departmental level (Figure 4). They discussed the dynamic and interdependent nature of the relationship between MI and online/offline learning processes leading to motor expertise.

They first reported an event-related synchronization of alpha and beta frequencies during the first instants following the MI onset stimuli in the Olympic athlete, usually reflecting neural inhibition and resting brain areas (Pfurtscheller, 1992; Neuper et al., 2006). They argued that the Olympic participant engaged in a kind of “reset phase” involving the occipital and parietal associative cortices, which is congruent with his subjective reports of absence of visual focus and *“empty mind*”. This phase appears very close from a meditative state of internal attentional focus (for a review see Aftanas and Golocheikine, 2001; Fell et al., 2010), and possibly allowed greater focus during forthcoming MI. Interestingly, the novice athlete did not report such use of contextualization strategies. Second, both participants exhibited an alpha desynchronization, but this comparable oscillatory pattern originated from the activation of very distinct neural networks. In the Olympic athlete, in addition to the bilateral precuneus activation emphasized for its role in the generation of motor images (Ogiso et al., 2000; Cavanna and Trimble, 2006), the desynchronization originated from premotor, primary sensorimotor and parietal activations. In the novice athlete, brain activations were more diffuse, and involved, in addition to associative parietal and occipital regions, the fusiform gyrus, which is emphasized for its role in online learning processes resulting from MIP interventions (Olsson et al., 2008c; Zhang et al., 2011). Overall, these data not only provide new insight about the time course of neural oscillations during MI, but also confirm that expertise is associated to a more focused recruitment of brain motor system regions during MI (for a review see Debarnot et al., 2014). By contrast, novices engage to a greater extent associative areas involved in the early phases of learning, and allocate a greater amount of mental resources to complete the MI task.

Historically, applied and fundamental MIP findings in healthy participants frequently provided a scientific rationale preceding clinical applications. Prompted by insights from Warner and McNeill (1988) (see also Decety, 1993), the number of clinical uses of MIP dramatically increased since the beginning of the 21th century (Di Rienzo et al., 2014). This attests an effective and positive transfer of MIP findings from sport sciences to clinical rehabilitation. Yet, this primarily concerns MIP findings related to online learning processes. Whether a greater understanding of MIP effects on offline learning processes (for instance at a fundamental level by determining the brain correlates of delayed performance gains) will contribute in the near future to the efficacy of clinical interventions represents a promising research issue. For instance, scheduling MIP sessions before/after periods of sleep could substantially boost the benefits and promote motor recovery. Likewise, whether current findings on online learning in healthy participants will also contribute to design effective MIP programs for clinical applications is a critical challenge. Considering the state-of-art in the field, extending our current understanding of: *(i)* the neurophysiological underpinnings of the individual predispositions to benefit from MIP; *(ii)* the relationship between MI ability and MIP effects on motor performance, assessed at behavioral and/or neurophysiological level; and *(iii)* the efficacy of combined MIP intervention (e.g., dynamic MI, action observation, etc., see “*Practical Implications*” Section) will have strong practical implications.

## Author Contributions

FDR, UD, SD, ES, CD, JD, CC and AG contributed to the conceptual background of the review and to the preparation of the manuscript (structure of the article, determination of the content of each section, search of information within the relevant literature). FDR, AG, UD, CC and JD wrote the article. FDR, UD, SD, ES, CD, JD, CC and AG all read and commented the article throughout the writing. They also reviewed the final article before submission.

## Conflict of Interest Statement

The authors declare that the research was conducted in the absence of any commercial or financial relationships that could be construed as a potential conflict of interest.

## Footnotes

^1^This result is unanimously supported by neurophysiological methods affording a high temporal resolution (e.g., magnetoencephalography, electroencephalography, transcranial magnetic stimulation), but less consistently reported in functional brain imaging experiments with a lower temporal resolution (e.g., functional magnetic resonance imaging, positron emission tomography).
